# Simultaneous solutions for first order and second order slips on micropolar fluid flow across a convective surface in the presence of Lorentz force and variable heat source/sink

**DOI:** 10.1038/s41598-019-51242-5

**Published:** 2019-10-11

**Authors:** K. Anantha Kumar, V. Sugunamma, N. Sandeep, M. T. Mustafa

**Affiliations:** 1 Department of Mathematics, Sri Venkateswara University, Tirupati, 517 502 India; 2 Department of Mathematics, Central University of Karnataka, Kalaburagi, 585 367 India; 3 Department of Mathematics, Statistics and Physics, Qatar University, Doha, 2713 Qatar

**Keywords:** Applied mathematics, Fluid dynamics

## Abstract

This report presents the flow and heat transfer characteristics of MHD micropolar fluid due to the stretching of a surface with second order velocity slip. The influence of nonlinear radiation and irregular heat source/sink are anticipated. Simultaneous solutions are presented for first and second-order velocity slips. The PDEs which govern the flow have been transformed as ODEs by the choice of suitable similarity transformations. The transformed nonlinear ODEs are converted into linear by shooting method then solved numerically by fourth-order Runge-Kutta method. Graphs are drowned to discern the effect of varied nondimensional parameters on the flow fields (velocity, microrotation, and temperature). Along with them the coefficients of Skin friction, couple stress, and local Nussel number are also anticipated and portrayed with the support of the table. The results unveil that the non-uniform heat source/sink and non-linear radiation parameters plays a key role in the heat transfer performance. Also, second-order slip velocity causes strengthen in the distribution of velocity but a reduction in the distribution of temperature is perceived.

## Introduction

Currently, the researchers and scientists have been focussed on the study of non-Newtonian fluid flow induced by stretched geometry, due to everyday desires of these assets in chemical and manufacturing practice. Corn flour, mud, syrups, dilatant, glue, chilli sauce, gypsum paste, body lotions, shampoo, toxoid vaccines, pasteurized milk, and soapy water are some industrial products included into this category. Various models are available in the data to review the flows of non-Newtonian fluids according to their adoptable essence. In which, the furthermost tackled liquids are micropolar shear thickening liquids. In 1964, the concept of micropolar shear thickening fluid was originated by Eringen^1^. Keeping this into mind, Rao and Rao^2^ explored the characteristics of micropolar liquid past a spherical geometry. Some notable information about the motion of non-Newtonian liquids via stretchable surface can be view in the earlier literature^3–5^. They acquired the problem solutions numerically with the aid of various finite difference schemes. The investigation on the problem of micropolar shear thickening fluid over a convectively heated surface can be view in ref.^6^. Waqas *et al*.^7^ scrutinized the essence of Biot number on forced convective non-Newtonian fluid flow induced by nonlinear stretchable surface. They stated that the micropolar constant has a propensity to regulate the rate of thermal transport. Recently, Lu *et al*.^8^ reported numerical scrutiny of the magnetohydrodynamic flow of shear thickening liquid across a stretched sheet in the attendance of radiation and chemical reaction.

The Phenomenon of stretching plays a decisive task in the examination of boundary layer flow owing to its incredible consequences in engineering applications such as polymer engineering, wire drawing, metallic beds cooling, glass forming approaches, hot rolling, plastic sheets extraction, paper production, etc. Based upon these applications the feature of the desired products will be controlled by the measure of thermal transfer. In 1970, the first study on the motion over a stretching surface was scrutinized by Crane^9^. Micropolar fluid motion induced by a strained surface was described by Chiam^10^. Hayat *et al*.^11^ reported the impact of Nusselt number on non-Newtonian liquid motion via a non-linear surface. The influence of organic response on the stagnated motion of Newtonian liquid across a cylinder was reported by Najib *et al*.^12^ and presented dual solutions for shrinking and stretching cases. Later, the work of Hayat *et al*.^11^ was then extended by Babu *et al*.^13^ with injection/suction. Soid *et al*.^14^ analyzed the heat transport attribute on time-dependent flow of Newtonian liquid induced by a shrinking sheet.

The analysis of magnetohydrodynamics has ample significances in the fields of cooling of the reactor, astrophysics, accelerators, design of heat exchangers, power generators, geophysics, plasma studies, and cancer research. Micropolar fluid motion across a nonlinear stretchable sheet under the impact of drag force was scrutinized by Hayat *et al*.^15^ and concluded that angular velocity has an inverse relationship with material parameter. Nadeem and Hussain^16^ explored the Lorentz force essence on viscous Newtonian fluid through the porous stretched surface. The MHD flow driven by a wedge or a cone with the aid of non-Fourier heat conduction was reported by Kumar *et al*.^17^ and concluded that temperature field is suppresses with a hike in the magnitude of relaxation parameter but enhances with magnetic field parameter. Impact of Lorentz force on forced convection Jeffery fluid through a permeable surface was reported by Ahmad and Ishak^18^. They implemented the finite difference numerical scheme to obtain the solution. The influence of thermophoresis and Brownian motion on MHD flow of nanoliquid across a solid surface in the attendance of linear Rossland approximation was investigated by Mabood *et al*.^19^. Mabood *et al*.^20^ presented an analytical solution for the problem of time-dependent flow of nanoliquid past a convectively heated surface in the attendance of Lorentz force and concluded that the magnetic field parameter has a proclivity to enhance the curves of heat function. The influence of variable viscosity on conducting flow of nanoliquid across a rotating surface in the presence of a porous medium was reported by Mabood *et al*.^21^. Recently, Kumar *et al*.^22^ investigated the flow characteristics of micropolar with radiation and frictional heating. They found that friction factor has an inverse relationship with the magnetic field parameter.

The impact of heat transport has sufficient significance in the sub-disciplines of paramedical and some engineering. Power creation, Oceanography, heat exchangers, magnetic drug targeting, thermal conduction in tissues, convection in earth’s mantle, electronic devices, boilers, missiles, fuel cells, etc are some applications of heat transfer. Polymer processing, gas turbines, production of paper, space vehicles, hypersonic fights, space technology, production of glass, gas cooled nuclear reactors are certain beneficial claims of radiation. Ziabakhsh *et al*.^23^ examined the heat generation and microrotation impacts on non-Newtonian liquid motion past a surface and concluded that a rise in blowing constant origins an improvement in the thermal fields. The influence of Newtonian heating on micropolar fluid motion driven by a stretched geometry was described by Qasim *et al*.^24^ and established that an augmentation in both the local Nusselt number and temperature distribution with larger Newtonian parameter. Cortell^25^ discussed the influence of the rate of heat transport on radiative liquid over a solid surface. Brownian motion influence on magnetohydrodynamic radiative flow of shear thickening liquid via stretchable geometry was reported by Farooq *et al*.^26^. Recently, the authors^27,28^ have paid their attention to investigate heat transport with nonlinear radiation.

The phenomenon of uneven heat sink/source has countless solicitations in medicine and many engineering happenings like cooling of metallic sheets, the intention of a thrust bearing, unpolished oil retrieval, etc. Pal^29^ studied the thermal transport attributes of time-dependent fluid flow across a stretched sheet with irregular heat sink/source. Some notable data about the irregular heat fall or raise effect on electrically conducting liquid flow due to stretching surface can be view in the ref.^30^. It was founded that uneven heat constraints have done a key character in the performance of thermal transport. RamReddy *et al*.^31^ numerically reported the behavior of Biot and Soret numbers on the forced convective motion of viscous liquid through a vertical plate and finalized that all the physical quantities have an inverse relationship. Patil *et al*.^32^ bestowed an inexact result for the forced convective motion subjected to the convective sheet. Kumar *et al*.^33^ presented the features of thermal transport on ferrofluid motion past a convective sheet with radiation. Impact of variable heat source/sink on MHD flow of micropolar liquid across a stretching surface with thermo-diffusion was reported by Mabood *et al*.^34^. The influence of heat source/sink and radiation on MHD flow of nanofluid past a nonlinear surface was scrutinized by Makinde *et al*.^35^ and Mabood *et al*.^36^ and concluded that the heat source/sink parameter has a tendency to enhance the temperature.

In the aforesaid investigations the impact of no slip condition is presumed. Mainly first order slip is important when the fluid is particulate like bubbles, mixtures (grease, egg yolk, the combination of oil and liquid), and polymer solutions. Many years ago, Navier^37^ recommended the momentum slip condition to liquid motion across a sheet. Behavior of secondary slip on Newtonian fluid flow due to a convective surface was analyzed by Fang *et al*.^38^. The impact of radiation on magnetohydrodynamic mixed convective slip motion was reported by Beg *et al*.^39^. Martin and Boyd^40^ investigated the influence of velocity slip on viscous liquid over a stretching sheet. With the aid of self-similar transformations, the flow equations are mutated and hence numerical results for the flow fields are presented. Attributes of slip motion and heat transport on magnetohydrodynamic micropolar liquid due to stretched sheet was reported by Ibrahim^41^. Recently, Mabood *et al*.^42^ scrutinized the influence of primary slip on boundary layer motion of radiative liquid across a melting surface. They noticed that higher values of the slip parameter cause an increment in the fluid temperature but the friction factor is inversely proportional to the slip parameter. Ibrahim *et al*.^43^ presented numerical scrutiny to examine the influence of chemical reaction and velocity slip on electrically conduction flow of non-Newtonian liquid across a convectively heated surface and found that Biot number has a propensity to heighten the heat transfer rate.

All the afore believed studies, the scientists put their struggles to gain the knowledge in the heat transfer of MHD flows under distinct physical aspects such as heat source/sink, linear Rossland approximation and slip effects, etc. but every few researchers deliberated the flow of micropolar shear thickening fluid in the appearance of nonlinear radiation and variable heat rise/fall effects. Most percentage of the research articles specified above, the authors used no slip or slip boundary conditions. But in the present study, we applied the second-order velocity slip condition to the boundary. Yet, no author delineated second-order velocity slip effects on MHD motion of micropolar liquid across a stretched surface with irregular heat sink/source. In the present article, we sighted at examining the nature of nonlinear radiation on MHD shear thickening fluid to fulfill the pre averred gap. R. K. based shooting application is applied to get the solution of higher ordered coupled ODEs. Results are divulged pictorially and presented numerical values for stipulated physical parameters.

## Mathematical Formulation

**Figure 1:**
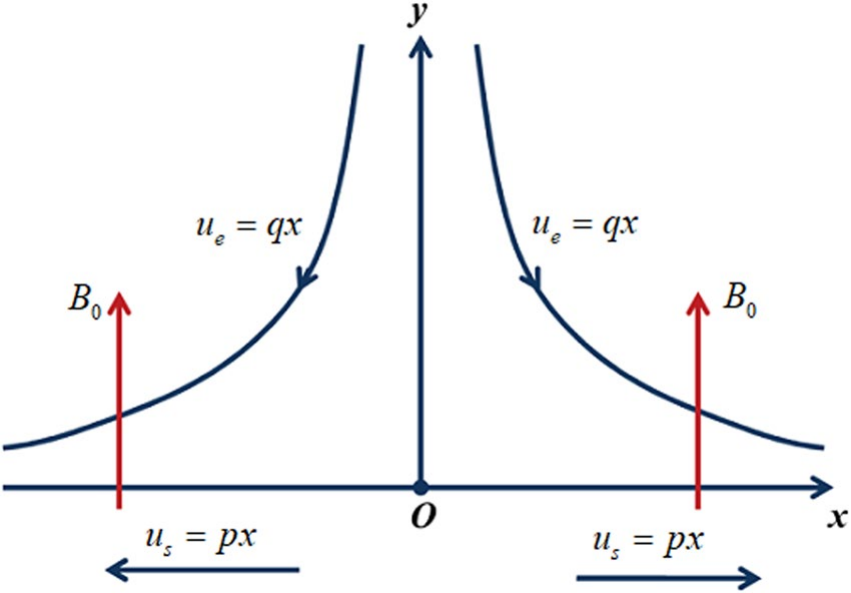
Flow Geometry.

Flow geometry of the model is exposed in Fig. 1. Let us imagine the two-dimensional flow of an incompressible, electrically accompanying micropolar fluid past a stretching sheet with the influence of secondary velocity slip. The fluid motion is laminar and time independent. Simultaneous solutions are presented for first and second order slips. The stretching sheet is pondered along the *x* route and *y* axis is perpendicular to it. Consider the velocities *u*_*s*_ = *px* and *u*_*e*_ = *qx*, where *p*, *q* are positive constants. The strength of the magnetic force *B*_0_ is exploited normal to the flow way as shown in Fig. 1. The following are some conventions on the present model.

- Micropolar liquid model.
- The impacts of uneven heat sink/source, nonlinear radiation are deemed.
- Influence of viscous dissipation is neglected.
- Convective and second-order velocity slip boundary conditions are employed.

With the above-declared assumptions, the flow equations will be (See refs^24,26,41^),

1 $$\frac{\partial u}{\partial x}+\frac{\partial v}{\partial y}=0,$$

2 $$\rho (u\frac{\partial u}{\partial x}+v\frac{\partial u}{\partial y})=\mu \frac{{\partial }^{2}u}{\partial {y}^{2}}-\kappa (\frac{\partial N}{\partial y}+\frac{{\partial }^{2}u}{\partial {y}^{2}})-\sigma {{B}_{0}}^{2}u,$$

3 $$\rho j(u\frac{\partial N}{\partial x}+v\frac{\partial N}{\partial y})=\varGamma \frac{{\partial }^{2}N}{\partial {y}^{2}}-\kappa (\frac{\partial u}{\partial y}+2N),$$

4 $$(\rho {C}_{P})(u\frac{\partial T}{\partial x}+v\frac{\partial T}{\partial y})=k\frac{{\partial }^{2}T}{\partial {y}^{2}}+q\prime\prime\prime -\frac{\partial {q}_{f}}{\partial y}$$

Here (*u*, *v*) are the constituents of velocity in the ways of (*x*, *y*) correspondingly, (*μ*, *ρ*) correspondingly, the viscosity (dynamic) and density, *κ* is the vortex viscosity, *N* is the microrotation velocity, electrical conductivity and micro-inertia density are $(\sigma ,j=\frac{\upsilon }{p})$ respectively, (*C*_*p*_, *k*) correspondingly, the heat capacitance and conductivity (thermal).

Consider

5 $$\Gamma =(\mu +\frac{\kappa }{2})j=\mu j(1+\frac{\alpha }{2})$$

where $\alpha =\frac{\kappa }{\mu }$ is the material parameter.

In Eq. (4) the second term in the R.H.S, $q\prime\prime\prime$ is defined as (See ref.^26^)

6 $$q\prime\prime\prime =\frac{k({T}_{s}-{T}_{\infty })\,{u}_{s}}{x\,\upsilon }({A}^{\ast }f^{\prime} +{B}^{\ast }\frac{(T-{T}_{\infty })}{({T}_{s}-{T}_{\infty })}),$$

Here *T*_*s*_ and *T*_∞_ are the nearby and ambient temperatures of the sheet correspondingly, the lessening and swelling values of A* and B* corresponds to heat fall or raise.

Consider

7 $${q}_{f}=\frac{-4{\sigma }^{\ast }}{3{k}^{\ast }}\frac{\partial {T}^{4}}{\partial y}=\frac{-16{\sigma }^{\ast }}{3{k}^{\ast }}{T}^{3}\frac{\partial T}{\partial y}$$

From Eqs (6 and 7), Eq. (4) converts as

8 $$(\rho {C}_{P})(u\frac{\partial T}{\partial x}+v\frac{\partial T}{\partial y})=k\frac{{\partial }^{2}T}{\partial {y}^{2}}+\frac{16{\sigma }^{\ast }}{3{k}^{\ast }}\frac{\partial }{\partial y}({T}^{3}\frac{\partial T}{\partial y})+\frac{k({T}_{s}-{T}_{\infty })\,{u}_{s}}{x\,\upsilon }({A}^{\ast }f^{\prime} +{B}^{\ast }\frac{(T-{T}_{\infty })}{({T}_{s}-{T}_{\infty })}),$$

Consider (See refs^28,41^),

9 $$v=0,u={u}_{s}+{u}_{{\rm{Slip}}},N=-\,{M}_{r}\frac{\partial u}{\partial y},\frac{\partial T}{\partial y}=\frac{{h}_{f}}{k}({T}_{s}-T),\,\,{\rm{at}}\,y=0,$$

10 $$u\to {u}_{e},N\to 0,T\to {T}_{\infty },\,\,{\rm{as}}\,y\to \infty ,$$

Consider the second-order velocity slip model (Ibrahim^41^)

11 $${u}_{{\rm{Slip}}}=\frac{2}{3}(\frac{3-a{l}^{2}}{a}-\frac{3}{2}\frac{1-{l}^{2}}{{K}_{n}})\lambda \frac{\partial u}{\partial y}-\frac{1}{4}({l}^{4}+\frac{2}{{K}_{n}^{2}}(1-{l}^{2})){\lambda }^{2}\frac{{\partial }^{2}u}{\partial {y}^{2}}=P\frac{\partial u}{\partial y}+Q\frac{{\partial }^{2}u}{\partial {y}^{2}},$$

12 $$l=\,\min (\frac{1}{{K}_{n}},1),$$

here *P*, *Q* are the constants, *K*_*n*_ is the Knudsen number, *λ* is the molecular free mean path, *a* is the coefficient of the momentum accommodation (0 ≤ *a* ≤ 1), *M*_*r*_ is a micro rotation parameter. From Eq. (12), we have 0 ≤ *l* ≤ 1, ∀*K*_*n*_. So *λ* is always non-negative. i.e., *Q* < 0 and hence the last term on R.H.S. of Eq. (11) is a positive number.

Here Knudsen number is the pivotal factor, which is a rate of the molecular free mean path of characteristic length. For very small *K*_*n*_ no-slip is noticed between the surface and fluid. However, *K*_*n*_ lies between 10^−3^ to 0.1, first order slip arises near the fluid-surface interaction.

Consider the transformations in order to get the dimensional less expressions of the flow equations: (See refs^24,26,41^),

13 $$\chi =\sqrt{p\upsilon }xf,\eta =\sqrt{\frac{p}{\upsilon }}y,N=px\,\sqrt{\frac{p}{\upsilon }}g,u=\frac{\partial \chi }{\partial y},v=-\frac{\partial \chi }{\partial x},T={T}_{\infty }(1+({\theta }_{w}-1)\theta ),{\theta }_{w}=\frac{{T}_{s}}{{T}_{\infty }},$$

Here *χ* is the stream function and *η* is the similarity variable, $f^{\prime} (\eta ),g(\eta )\& \theta (\eta )$ are the dimensionless flow fields and *θ*_*w*_ is the temperature ratio parameter.

From Eq. (13), Eq. (1) satisfied trivially and the Eqs (2), (3) and (8) becomes

14 $$(1+\alpha )\frac{{d}^{3}f}{d{\eta }^{3}}+f\frac{{d}^{2}f}{d{\eta }^{2}}-{(\frac{df}{d\eta })}^{2}+\alpha \frac{dg}{d\eta }-M\frac{df}{d\eta }=0,$$

15 $$(1+\frac{\alpha }{2})\frac{{d}^{2}g}{d{\eta }^{2}}+f\frac{dg}{d\eta }-g\frac{df}{d\eta }-\alpha (2g+\frac{{d}^{2}f}{d{\eta }^{2}})=0,$$

16 $$\begin{array}{c}\frac{{d}^{2}\theta }{d{\eta }^{2}}+f\Pr \frac{d\theta }{d\eta }+({A}^{\ast }\frac{df}{d\eta }+{B}^{\ast }\theta )+\\ {N}_{r}(\begin{array}{c}\frac{{d}^{2}\theta }{d{\eta }^{2}}+{(\theta )}^{3}\frac{{d}^{2}\theta }{d{\eta }^{2}}{({\theta }_{w}-1)}^{3}+3{(\theta )}^{2}{(\frac{d\theta }{d\eta })}^{2}{({\theta }_{w}-1)}^{3}+\\ 3{(\theta )}^{2}\frac{{d}^{2}\theta }{d{\eta }^{2}}{({\theta }_{w}-1)}^{2}+6\theta {(\frac{d\theta }{d\eta })}^{2}{({\theta }_{w}-1)}^{2}+3\theta \frac{{d}^{2}\theta }{d{\eta }^{2}}({\theta }_{w}-1)+3{(\frac{d\theta }{d\eta })}^{2}({\theta }_{w}-1)\end{array})=0,\end{array}$$

The corresponding mutated boundary conditions are

17 $$\frac{df}{d\eta }=1+\gamma \frac{{d}^{2}f}{d{\eta }^{2}}+\delta \frac{{d}^{3}f}{d{\eta }^{3}},f=0,g=-\,{M}_{r}\frac{{d}^{2}f}{d{\eta }^{2}},\frac{d\theta }{d\eta }=-\,Bi(1-\theta )\,\,at\,\,\eta =0,$$

18 $$\frac{df}{d\eta }\to \lambda ,g\to 0,\theta \to 0\,\,as\,\,\eta \to \infty ,$$

here $\alpha ,M,\Pr ,{N}_{r},Bi,\lambda ,\gamma \& \delta$ are the micropolar parameter, magnetic field parameters, Prandtl number, nonlinear radiation parameter, Biot number, stretching ratio parameter, first and second order slip parameter respectively and these are defined by

19 $$M=\frac{\sigma {B}_{0}^{2}}{p\,\rho },\Pr =\frac{\mu {C}_{p}}{k},{N}_{r}=\frac{16{\sigma }^{\ast }{T}_{\infty }^{3}}{3k{k}^{\ast }},\gamma =P\sqrt{\frac{p}{\upsilon }}( > 0),\delta =Q\frac{p}{\upsilon }( < 0),Bi=\frac{{h}_{f}}{k}\sqrt{\frac{\upsilon }{p}},\lambda =\frac{q}{p},$$

Calculated friction factor, couple stress coefficient and heat transfer rate are

20 $$\begin{array}{c}{\mathrm{Re}}_{x}^{1/2}{C}_{F}=2(1+\alpha (1-{M}_{r})){(\frac{{d}^{2}f}{d{\eta }^{2}})}_{\eta =0},{C}_{S}=(1+\frac{\alpha }{2}){(\frac{dg}{d\eta })}_{\eta =0},\\ {\mathrm{Re}}_{x}^{-1/2}Nu=-\,(1+{N}_{r}{({\theta }_{w})}^{3}){(\frac{d\theta }{d\eta })}_{\eta =0},\end{array}\}$$

where ${\mathrm{Re}}_{x}=\frac{p\,{x}^{2}}{\upsilon }$ is the confined Reynolds number.

## Deliberation of Results

This section sightsees the inspiration of flow variable parameters on the flow fields. The scheme of nonlinear and coupled ODEs (14)–(16) with the restrictions of the boundary (17)–(18) have resolved numerically with the sequential solicitation of shooting and R.K. methods. The effect of varied dimensionless parameters on the fluid temperature, micro-rotation, and velocity field is exposed via plots. Further, we scrutinize the influence of the same variables on the physical quantities and the consequences are exhibited in the table. We prescribed the values of $\alpha =2,M=1,{\theta }_{w}=0.5,{N}_{r}=0.3,{A}^{\ast }={B}^{\ast }=0.2,\Pr =7,Bi=0.5,\gamma =1.0$ and *λ* = 0.2 for computation purpose. In the pictures, solid lines stipulate the impact of first-order slip and dashed lines stipulates the impact of second-order slip.

For the verification of accuracy, the present results of friction factor (*C*_*F*_) are compared with the results obtained by Ibrahim^41^ through Table 1. This table shows that the present results are in good agreement with the results reported by Ibrahim^41^ for ${N}_{r}=0,{\theta }_{w}=0,$ ${A}^{\ast }=0,{B}^{\ast }=0,\gamma =1,\delta =-\,1,Bi=0,\Pr =1$ and ${M}_{r}=0.5$.

**Table 1:** Comparison of friction factor (*C*_*F*_) for different values of *α* and *M* when $${N}_{r}=0,$$ $${\theta }_{w}=0,{A}^{\ast }=0,$$$${B}^{\ast }=0,\gamma =1,\delta =-\,1,Bi=0,\Pr =7$$ and $${M}_{r}=0.5$$.

*α*	*M*	Ibrahim^41^	Present study
1	0.2	0.3173	0.31709
2	0.2	0.3068	0.30676
3	0.2	0.2971	0.29713
4	0.2	0.2884	0.28841
0.1	0.1	0.3220	0.32196
0.1	0.2	0.3262	0.32623
0.1	0.3	0.3293	0.32933

The influence of relevant parameters on $f^{\prime\prime} (0),g^{\prime} (0)$ and $-\theta ^{\prime} (0)$ for first and second order slips of micropolar shear-thickening liquid flow through a stretched surface is sightseen by Tables 2 and 3. From the table, it is noticed that a rise in the values of Lorentz force results a drop in all the quantities ($f^{\prime\prime} (0),g^{\prime} (0)$ and $-\theta ^{\prime} (0)$) however a reverse consequence is perceived for material parameter. Swelling values of micropolar constant upshots a hike in $g^{\prime} (0)$ but a reduction is noticed for a larger magnetic field parameter. Larger *M*_*r*_ yields an increment in the couple stress coefficient, friction factor, and the measure of heat transport. Also, the measure of thermal transport is supreme for larger *Bi* for both the cases. Heat generation or absorption and temperature ratio parameters cause a reduction in heat transfer rate while an opposite outcome is noticed with nonlinear radiative energy for both the slips (first and second order) cases. From Table 3 it is noticed that an increase in *γ* results a hike in both $f^{\prime\prime} (0),g^{\prime} (0)$ but a contrary development is noticed for second order slip parameter. Also, the thermal transport rate is a cumulative function of second order slip parameter but an inverse outcome is discerned for *γ*.

**Table 2:** Influence of sundry flow parameters on *C*_*F*_, *C*_*S*_ and *Nu* for *δ* = 0 and *δ* = 1.

	*C*_*F*_		*C*_*S*_		*Nu*	
	*δ* = 0	*δ* = 1	*δ* = 0	*δ* = 1	*δ* = 0	*δ* = 1
*M* = 1.0	−0.3155	−0.3463	0.0588	0.0641	0.3123	0.3176
*M* = 2.0	−0.3429	−0.3923	0.0515	0.0584	0.2880	0.2989
*M* = 3.0	−0.3594	−0.4265	0.0458	0.0538	0.2601	0.2801
*α* = 1.0	−0.6004	−0.8207	−0.3068	−0.4296	0.2734	0.3003
*α* = 2.0	−0.5669	−0.7251	−0.2752	−0.3585	0.2891	0.3072
*α* = 3.0	−0.5407	−0.6648	−0.2460	−0.3072	0.2983	0.3120
*M*_*r*_ = 1.0	−0.2703	−0.2767	0.5958	0.6101	0.2795	0.2808
*M*_*r*_ = 2.0	−0.2391	−0.2302	0.6328	0.6822	0.2916	0.2898
*M*_*r*_ = 3.0	−0.2154	−0.1974	0.7326	0.7674	0.2987	0.2949
*N*_*r*_ = 1.0	−0.3945	−0.4314	−0.0998	0.1113	0.3481	0.3508
*N*_*r*_ = 2.0	−0.3945	−0.4314	−0.0998	0.1113	0.3456	0.3479
*N*_*r*_ = 3.0	−0.3945	−0.4314	−0.0998	0.1113	0.3429	0.3451
*θ*_*w*_ = 1.0	−0.3957	−0.4324	−0.0994	−0.1107	0.3361	0.3399
*θ*_*w*_ = 3.0	−0.3957	−0.4324	−0.0994	−0.1107	0.3212	0.3250
*θ*_*w*_ = 5.0	−0.3957	−0.4324	−0.0994	−0.1107	0.3201	0.3233
A* = 0.1	−0.3957	−0.4324	−0.0994	−0.1107	0.3589	0.3622
A* = 0.3	−0.3957	−0.4324	−0.0994	−0.1107	0.3398	0.3428
A* = 0.5	−0.3957	−0.4324	−0.0994	−0.1107	0.3207	0.3234
B* = 0.0	−0.3957	−0.4324	−0.0994	−0.1107	0.3544	0.3572
B* = 1.0	−0.3957	−0.4324	−0.0994	−0.1107	0.3224	0.3281
B* = 2.0	−0.3957	−0.4324	−0.0994	−0.1107	0.2598	0.2741
*Bi* = 0.5	−0.4080	−0.4438	−0.0968	−0.1076	0.0880	0.0882
*Bi* = 1.0	−0.4080	−0.4438	−0.0968	−0.1076	0.1647	0.1654
*Bi* = 1.5	−0.4080	−0.4438	−0.0968	−0.1076	0.2327	0.2342

**Table 3:** Impact of second and first order slip parameters on *C*_*F*_, *C*_*S*_ and *Nu*.

	*C*_*F*_	*C*_*S*_	*Nu*
*δ* = 0.1	−0.6934	−0.8149	0.1371
*δ* = 0.3	−0.8641	−1.0267	0.1412
*δ* = 0.5	−1.2135	−1.4691	0.1468
*γ* = 0.1	−1.8403	−2.2850	0.1536
*γ* = 0.3	−1.1360	−1.3707	0.1455
*γ* = 0.5	−0.8607	−1.0234	0.1407

**Figure 2:**
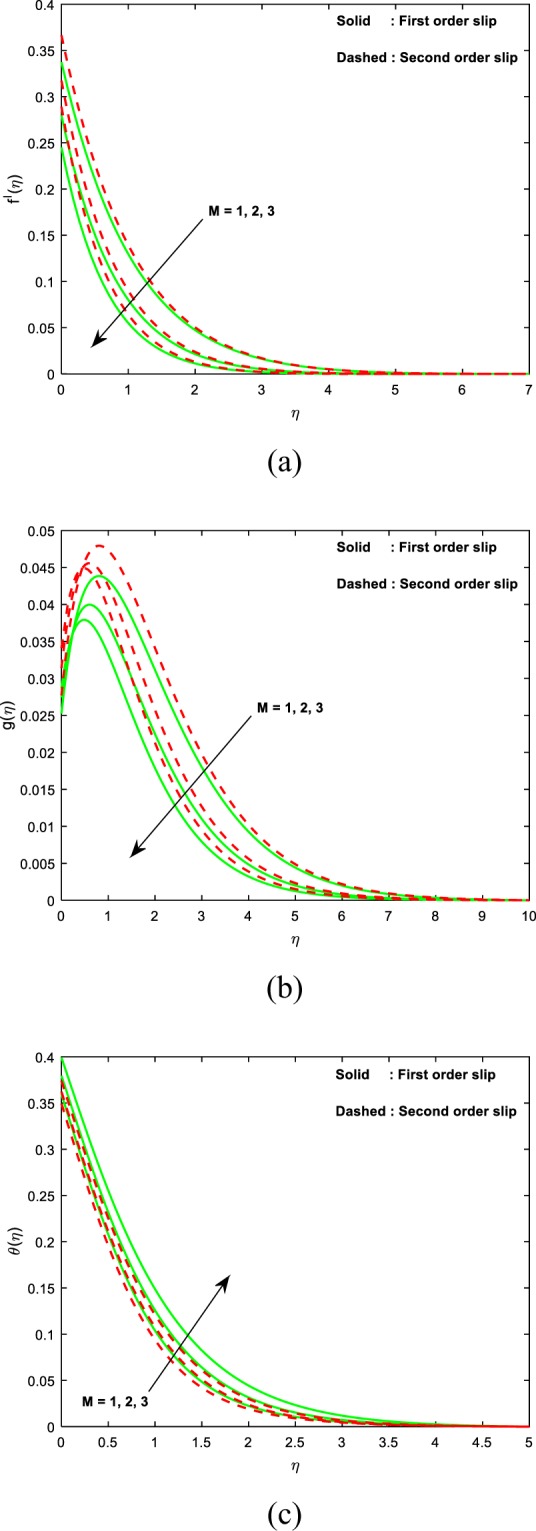
Impact of *M* on (**a**) velocity (**b**) microrotation (**c**) temperature.

Figure 2 renders for the variation of magnetic field parameter (*M*) on the velocity, microrotation and thermal fields. Figure 2(a),(b) divulge that, both the distributions of linear and angular momentum suppresses as the values of magnetic field variable enhances. This contest the physical interpretation on hiring the magnetic force to an electrically conducting fluid, and this provides an elevation in the drag force, which consequences in the decelerating strength on velocities. Owing to this, a reduction in the fields of velocity and microrotation is noticed. But in case of thermal fields, it’s quite reverse to fluid velocities as exposed in Fig. 2(c). From the plots, it is spotted that both the velocity and the angular velocity are attained maximum due to second order slip and the maximum temperature is noticed for first order slip.

**Figure 3:**
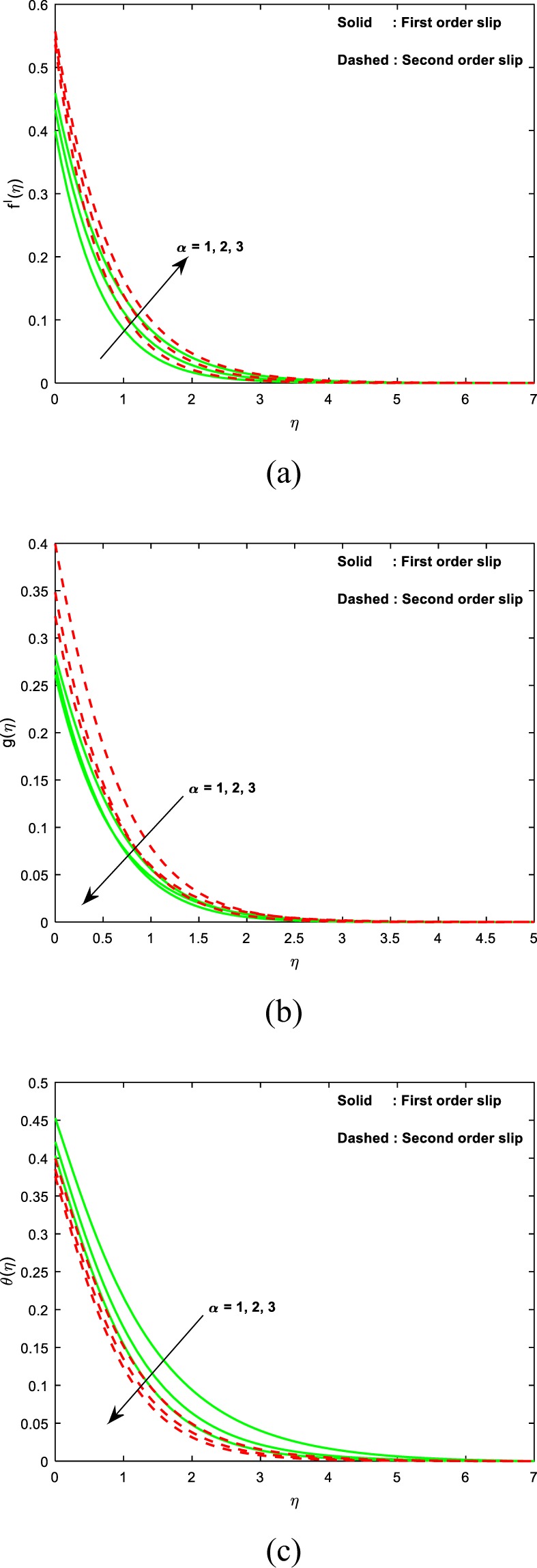
Impact of *α* on (**a**) velocity (**b**) microrotation (**c**) temperature.

Figure 3 is plotted to know the essence of the micropolar parameter (*α*) on the velocity, microrotation and thermal fields. It is interesting to note that, an enhancement in the values of *α* boosts the linear velocity, however, a contrary consequence is detected for microrotation and thermal fields. The results specify that the momentum transfer layer-by-layer is boosted expressively owing to the escalation of viscosity caused by the collective micro-rotation of particles, i.e., large values of the micropolar parameter; on the other hand, the thermal diffusions are weakened slightly. Hence, there is a reduction in both the micro-rotation and thermal fields are noticed for larger material constant.

**Figure 4:**
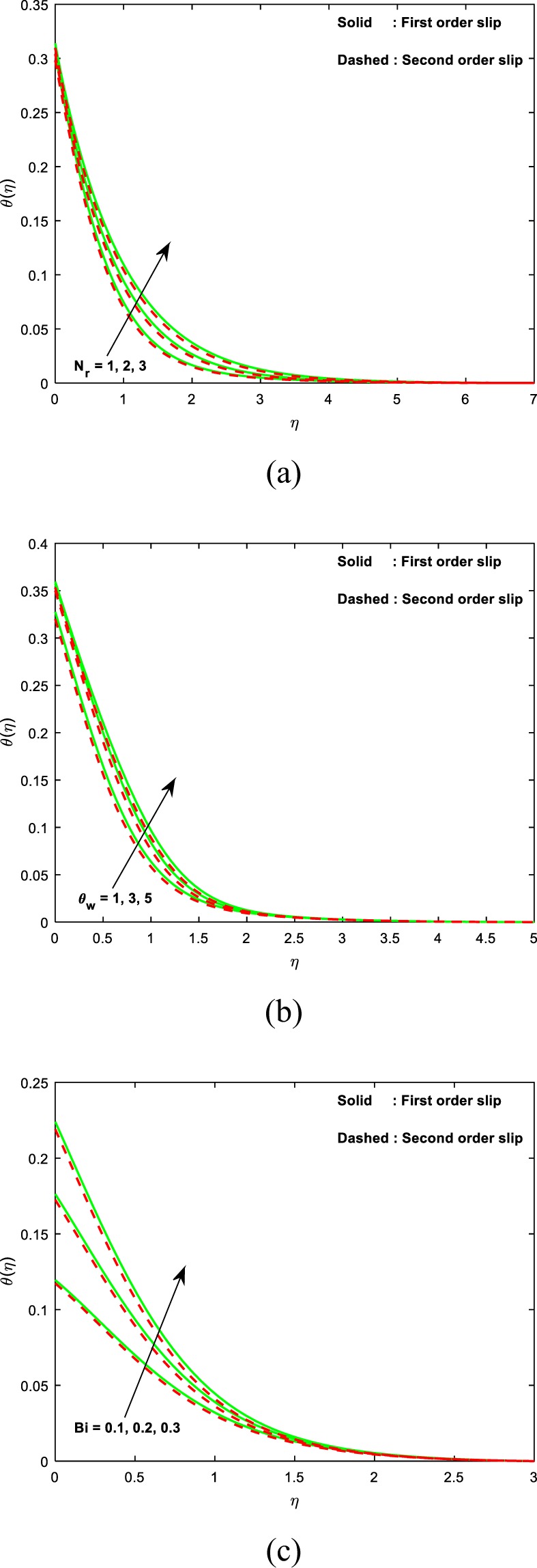
Curves of temperature with the variant in (**a**) *N*_*r*_ (**b**) *θ*_*w*_ (**c**) *Bi*.

Figure 4(a) is deliberate to discuss the influence of radiation parameter (*N*_*r*_) on the distribution of temperature. From the figure, it is noticed that the impression of radiation parameter *N*_*r*_ on θ(*η*) is increasing. It is familiar that the mechanism of radiation and is the heat transference phenomenon which releases the energy via fluid particles such that some additional heat is produced in the flow. It is worth mention that the influence of radiation becomes more significant as ${N}_{r}\to \infty$ and the influence of radiation can be neglected when ${N}_{r}=0$. Moreover, high heat transfer is attained in the presence of first-order slip than that of second-order slip.

The essence of the temperature ratio parameter on fluid temperature is investigated through Fig. 4(b). It is worth mention that thermal field enhances with increasing values of *θ*_*w*_. Mathematically, ${\theta }_{w}=\frac{{T}_{w}}{{T}_{\infty }}$ is the ratio of temperature at the surface to the temperature at a free stream. The value of *θ*_*w*_ must be greater than 1 for the nonlinear radiation. Also, an increase in temperature ratio parameter causes a hike in the temperature along the surface. As a result, the distribution of thermal field and the corresponding layer thickness enhances. It is significant to remark that as ${\theta }_{w}\to 1$, the temperature of nonlinear Rosseland and linear Rosseland approximation are the same. We observed an interesting result that the distribution of temperature ($\theta (\eta )$) is high for $\delta =0$ when compared to that of $\delta \ne 0$.

The influence of Biot number (*Bi*) on the fluid temperature is investigated through Fig. 4(c). We see that the ascending values of the Biot number improve the fluid temperature. Biot number occurs in the current investigation owing to the assumption of convective boundary condition and signifies the ratio of diffusive resistance within the sheet to the convective resistance at the surface of the sheet. Thus smaller values of the Biot number provides high convective resistance at the surface, which leads to low heat transfer rate from the sheet to the fluid. Hence the fluid temperature is an increasing function of Biot number. We observed a motivating consequence that the distribution of temperature (*θ*(*η*)) is high for *δ* = 0 when compared that of *δ* ≠ 0.

**Figure 5:**
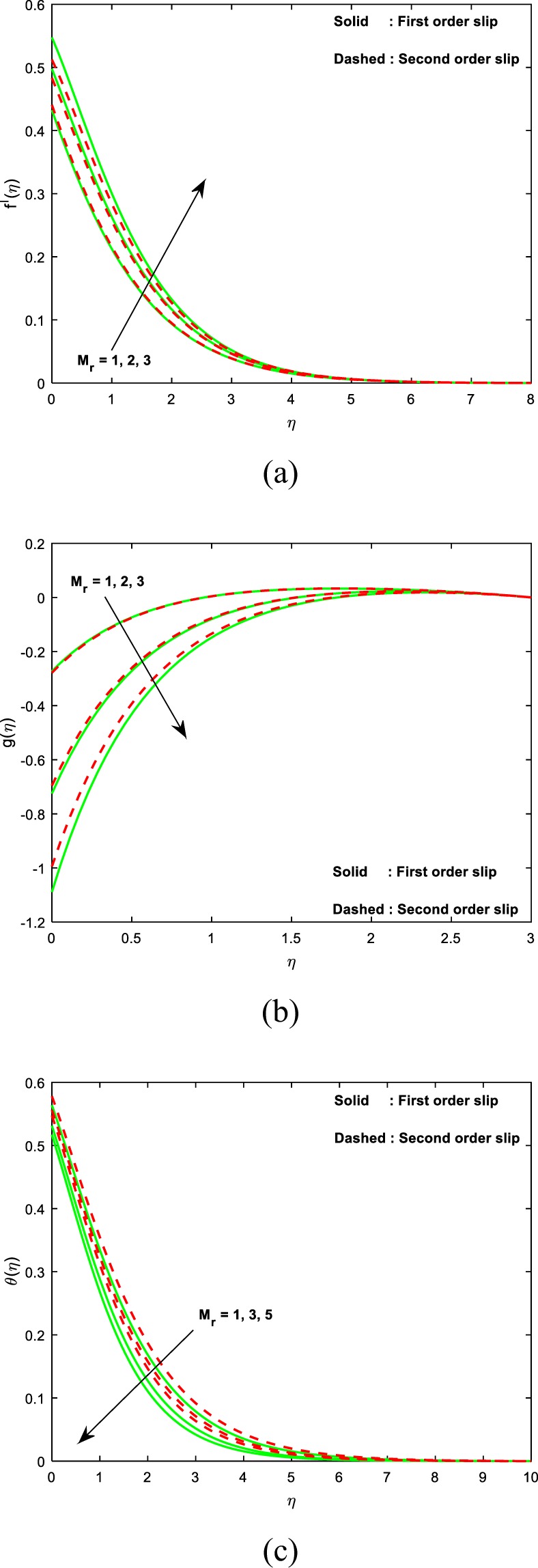
Impact of *M*_*r*_ on (**a**) velocity (**b**) microrotation (**c**) temperature.

Figure 5 is sketched to know the influence of microrotation parameter on the velocity, microrotation and thermal fields. From Fig. 5(a), it is spotted that fluid velocity is an increasing function of the microrotation parameter. As a result, we glimpse a decrement in temperature and microrotation profiles from Fig. 5(b),(c) respectively.

**Figure 6:**
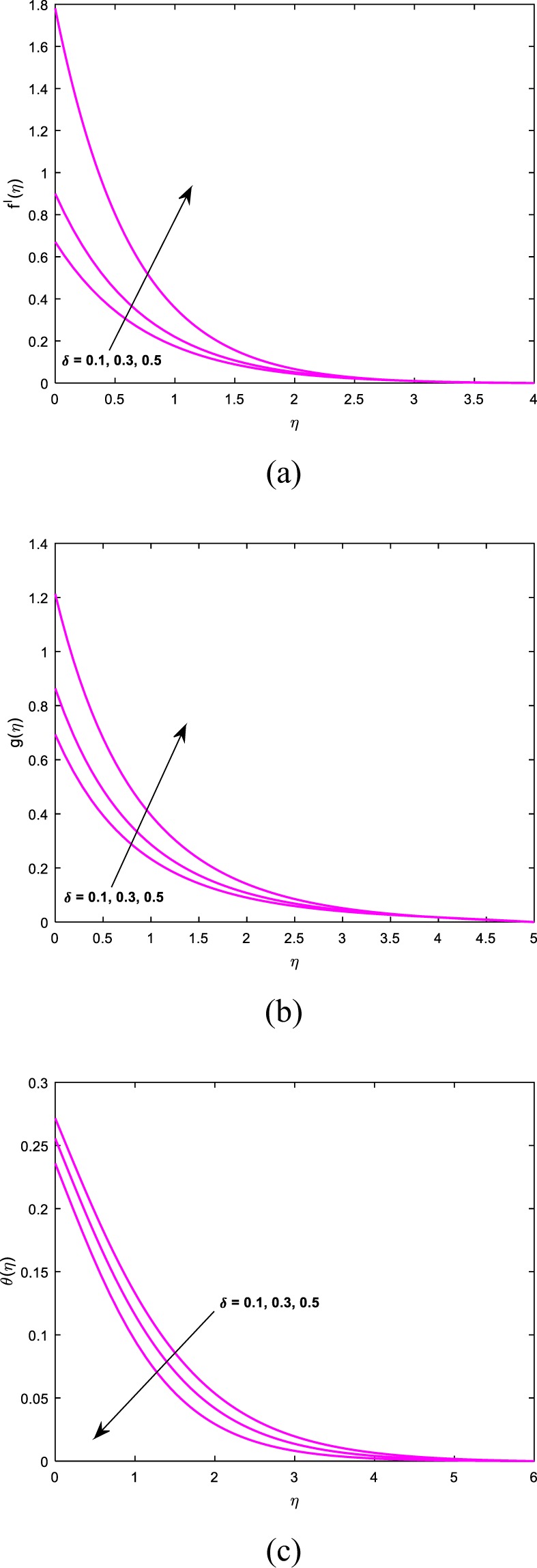
Impact of *δ* on (**a**) velocity (**b**) microrotation (**c**) temperature.

**Figure 7:**
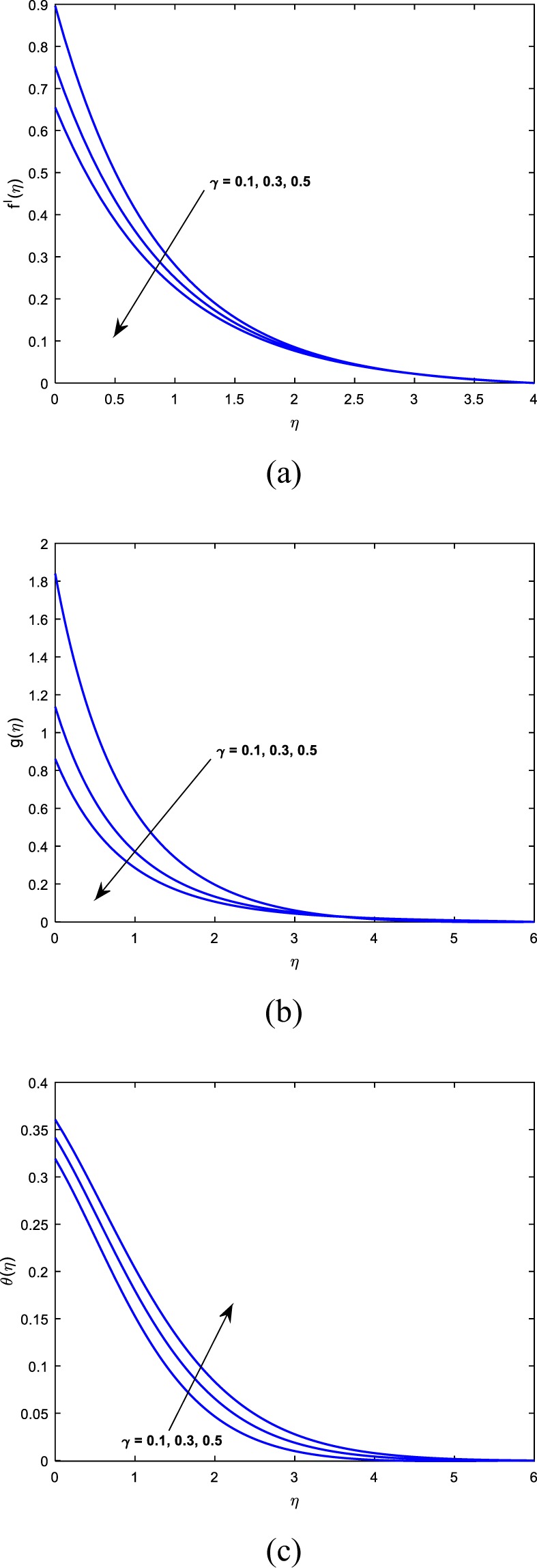
Impact of *γ* on (**a**) velocity (**b**) microrotation (**c**) temperature.

Figures 6 and 7 are drawn to see the influence of *γ* and *δ* on $f^{\prime} (\eta ),g(\eta )$ and $\theta (\eta )$ respectively. From Fig. 6(a),(b), we eye that an accelerating values of *δ* results a hike in dimensionless velocity but the thermal field is a decelerating function of *δ*. Hence Fig. 6(c) says that the distribution of angular momentum increases with an increment in *δ*. The impression of *γ* on $\theta (\eta ),g(\eta )$ and $f^{\prime} (\eta )$ is investigated via Fig. 7(a)–(c) respectively. For increasing values of *γ* results a hike in both the thermal field and the corresponding boundary thickness. Moreover, the fluid velocity and microrotation profiles are decelerating functions of *γ*. So we conclude that the primary and the secondary slip parameters are proportional to each other. Hence the maximum temperature is noticed for second-order slip and maximum velocity is noticed for first-order slip parameter.

**Figure 8:**
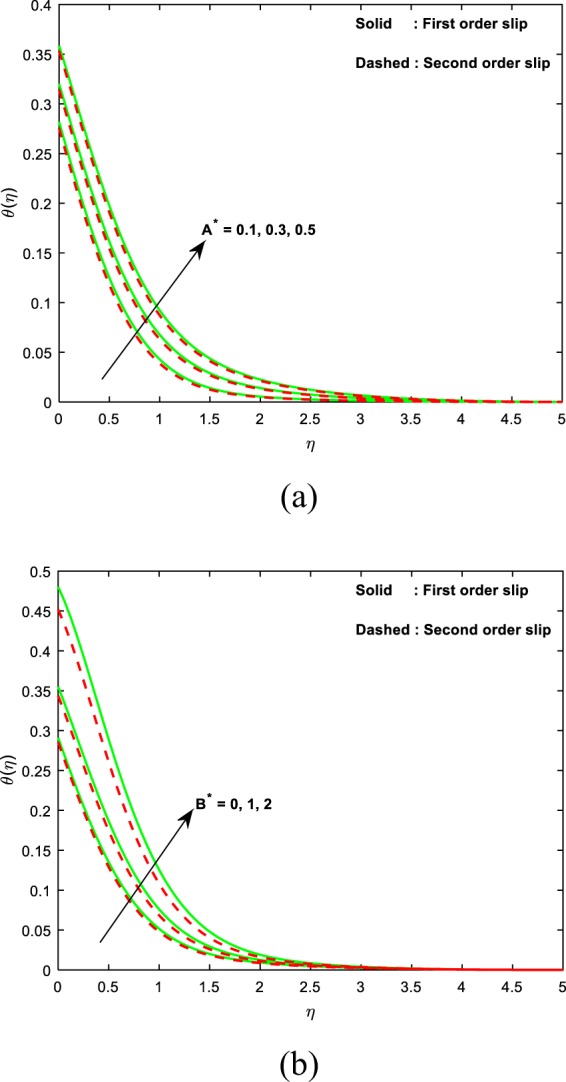
Curves of temperature with the variant in (**a**) A* (**b**) B*.

Figure 8(a),(b) reveal the nature of fluid temperature for different values of non-uniform heat source/sink parameters (A*, B*). It is detected that swelling values of irregular heat source/sink parameters results a hike in the fluid temperature. Actually, an increasing values of irregular heat parameters act as an agent to produce temperature in the flow. Due to this, we observed that a rise in the fluid temperature for swelling values of A*, B*. It is prominent that the more heat transfer is attained in the absence of second-order slip.

## Findings of the Problem

This paper narrates the flow and thermal transport attributes of shear thinking fluid across a stretching surface with Biot number and drag force. Simultaneous solutions were presented for first and second order slips. The principal outcomes are listed below

- Microrotation profile is a decelerating function of micropolar parameter.
- A rise in the microrotation parameter causes the same in *g*(*η*) and *θ*(*η*) but a reverse trend is noticed for $f^{\prime} (\eta )$.
- Fluid temperature is enhanced due to an increment in the values of both *N*_*r*_ and *θ*_*w*_.
- The Velocity distribution upturns for rising values of magnetic field parameter.
- In all the figures supreme velocity is achieved due to second order velocity slip however the supreme temperature is achieved due to first order slip.
- The irregular heat raise/fall parameters plays key role in the heat transfer performance.
- The nature of all the physical quantities is reverse for slip parameters.

## References

[1] Eringen AC (1964). Simple microfluids. Int. J. Eng. Sci..

[2] Rao SKL, Rao PB (1970). The slow stationary flow of micropolar liquid past a sphere. J. Eng. Mech..

[3] Agarwal RS, Bhargava R, Balaji AVS (1989). Finite element solution of flow and heat transfer of a micropolar fluid over a stretching sheet. Int. J. Eng. Sci..

[4] Bhargava R, Kumar L, Takhar HS (2003). Finite element solution of mixed convection micropolar flow driven by a porous stretching sheet. Int. J. Eng. Sci..

[5] Eldabe NT, Elshehawey EF, Elbarbary EME, Elgazery NS (2005). Chebyshev finite difference method for MHD flow of a micropolar fluid past a stretching sheet with heat transfer. Appl. Math. Comp..

[6] Anantha Kumar K, Sugunamma V, Sandeep N (2018). Numerical exploration of MHD radiative micropolar liquid flow driven by stretching sheet with primary slip: a comparative study. J. Non-Equilib. Thermodyn..

[7] Waqas M (2016). Magnetohydrodynamic (MHD) mixed convective flow of micropolar liquid due to non-linear stretched sheet with convective condition. Int. J. Heat Mass Transf..

[8] Lu, D., Ramzan, M., Ahmad, S., Chung, J. D. & Farooq, A. A numerical treatment of MHD radiative flow of Micropolar nanofluid with homogeneous-heterogeneous reactions past a nonlinear stretched surface. *Sci. Rep*. **8**, 10.1038/s41598-018-30965-x (2018). 10.1038/s41598-018-30965-x PMC6102272 30127369

[9] Crane LJ (1970). Flow past a stretching plate. J. Appl. Math. Phys. (ZAMP).

[10] Chiam TC (1982). Micropolar fluid flow over a stretching sheet. ZAMM.

[11] Hayat T, Abbas Z, Javed T (2008). Mixed convection flow of a micropolar fluid over a non linearly stretching sheet. Phys. Lett. A.

[12] Najib, N., Bachok, N., Arifin, N. M. & Ishak, A. Stagnation point flow and mass transfer with chemical reaction past a stretching/shrinking cylinder. *Sci. Rep*. **4**, 10.1038/srep04178 (2014). 10.1038/srep04178 PMC3935189 24569547

[13] Babu MJ, Raju CSK, Sandeep N (2015). Stagnation point flow of a micropolar fluid over a nonlinearly stretching surface with suction. Int. J. Sci. Eng. Res..

[14] Soid SK, Ishak A, Pop I (2017). Unsteady MHD flow and heat transfer over a shrinking sheet with ohmic heating. Chin. J. Phys..

[15] Hayat T, Javed T, Abbas Z (2009). MHD flow of a micropolar fluid near a stagnation-point towards a non-linear stretching surface. Nonlinear Anal. Real World Appl..

[16] Nadeem S, Hussain SN (2014). Heat transfer analysis of Williamson fluid over exponentially stretching surface. Appl. Math. Mech..

[17] Kumar KA, Reddy JVR, Sugunamma V, Sandeep N (2018). Magnetohydrodynamic Cattaneo-Christov flow past a cone and a wedge with variable heat source/sink. Alex. Eng. J..

[18] Ahmad K, Ishak A (2017). Magnetohydrodynamic (MHD) Jeffery fluid over a stretching vertical surface in a porous medium. Prop. Power Res..

[19] Mabood F, Ibrahim SM, Khan WA (2016). Framing the features of Brownian motion and thermophoresis on radiative nanofluid flow past a rotating stretching sheet with magnetohydrodynamics. Res. Phys..

[20] Mabood F, Khan WA (2016). Analytical study for unsteady nanofluid MHD Flow impinging on heated stretching sheet. J. Mol. Liq..

[21] Mabood F, Khan WA, Makinde OD (2017). Hydromagnetic flow of a variable viscosity nanofluid in a rotating permeable channel with hall effects. J. Eng. Thermophys..

[22] Kumar KA, Sugunamma V, Sandeep N (2018). Impact of non-linear radiation on MHD non-aligned stagnation point flow of micropolar fluid over a convective surface. J. Non-Equilib. Thermodyn.

[23] Ziabakhsh Z, Domairry G, Bararnia H (2009). Analytical solution of non-Newtonian micropolar fluid flow with uniform suction/blowing and heat generation. J. Taiwan Inst. Chem. Eng..

[24] Qasim, M., Khan, I. & Shafie, S. Heat transfer in a micropolar fluid over a stretching sheet with Newtonian heating. Plos One **8**, Article Id: e59393 (2008). 10.1371/journal.pone.0059393 PMC3614560 23565151

[25] Cortell R (2014). Fluid flow and radiative nonlinear heat transfer over a stretching sheet. J. King Saud Uni. Sci..

[26] Farooq M (2016). MHD stagnation point flow of viscoelastic nanofluid with nonlinear radiation effects. J. Mol. Liq..

[27] Babu MJ, Sandeep N, Raju CSK, Reddy JVR, Sugunamma V (2016). Nonlinear thermal radiation and induced magnetic field effects on stagnation-point flow of ferrofluids. J. Adv. Phys..

[28] Ramandevi B, Reddy JVR, Sugunamma V, Sandeep N (2017). Combined influence of viscous dissipation and non-uniform heat source/sink on MHD non-Newtonian fluid flow with Cattaneo-Christov heat flux. Alex. Eng. J..

[29] Pal D (2011). Combined effects of non-uniform heat source/sink and thermal radiation on heat transfer over an unsteady stretching permeable surface. Commun. Nonlinear Sci. Numer. Simulat..

[30] Reddy JVR, Anantha Kumar K, Sugunamma V, Sandeep N (2017). Effect of cross diffusion on MHD non-Newtonian fluids flow past a stretching sheet with non-uniform heat source/sink: A comparative study. Alex. Eng. J..

[31] RamReddy C, Murthy PVSN, Chamkha AJ, Rashad AM (2013). Soret effect on mixed convective flow in a nanofluid under convective boundary condition, Int. J. Heat Mass Transf..

[32] Patil PM, Momoniat E, Roy S (2014). Influence of convective boundary condition on double diffusive mixed convection from permeable vertical surface. Int. J. Heat Mass Transf..

[33] Kumar KA, Reddy JVR, Sugunamma V, Sandeep N (2017). Impact of frictional heating on MHD radiative ferrofluid past a convective shrinking surface. Def. Diff. Forum.

[34] Mabood F, Ibrahim SM, Rashidi MM, Shadloo MS, Lorenzini G (2016). Non-uniform heat source/sink and Soret effects on MHD non-Darcian convective flow past a stretching sheet in a micropolar fluid with radiation. Int. J. Heat Mass Transf..

[35] Makinde OD, Mabood F, Ibrahim MS (2018). Chemically reacting on MHD boundary-layer flow of nanofluids over a non-linear stretching sheet with heat source/sink and thermal radiation. Therm. Sci..

[36] Mabood, F., Ibrahim, S. M. & Khan, W. A. Effect of melting and heat generation/absorption on Sisko nanofluid over a stretching surface with nonlinear radiation. *Phys. Scrip*. **94**, Artile ID: 065701, (2019).

[37] Navier CLM (1827). Sur les lois du mouvement des fluides. Mem. Acad. Royal Sci. Inst. Fr.

[38] Fang T, Yao S, Zhang J, Aziz A (2010). Viscous flow over a shrinking sheet with a second order slip flow model. Commun. Nonlinear Sci. Number Simul.

[39] Beg OA, Uddin MJ, Rashidi MM, Kavyani N (2014). Double-diffusive radiative magnetic mixed convective slip flow with Biot and Richardson number effects. J. Eng. Therm..

[40] Martin MJ, Boyd ID (2006). Momentum and heat transfer in a laminar boundary layer with slip flow. J. Therm. Heat Transf..

[41] Ibrahim W (2017). MHD boundary layer flow and heat transfer of micropolar fluid past a stretching sheet with second order slip. J. Braz. Soc. Mech. Sci. Eng..

[42] Mabood F, Shafiq A, Hayat T, Abelman S (2017). Radiation effects on stagnation point flow with melting heat transfer and second order slip. Res. Phys..

[43] Ibrahim SM, Kumar PV, Lorenzini G, Lorenzini E, Mabood F (2017). Numerical study of the onset of chemical reaction and heat source on dissipative MHD stagnation point flow of Casson nanofluid over a nonlinear stretching sheet with velocity slip and convective boundary conditions. J. Eng. Thermophys..

